# Transfection Technologies for Next-Generation Therapies

**DOI:** 10.3390/jcm14155515

**Published:** 2025-08-05

**Authors:** Dinesh Simkhada, Su Hui Catherine Teo, Nandu Deorkar, Mohan C. Vemuri

**Affiliations:** Biopharma and Bioproduction, Cell and Gene Therapy, Avantor, 77 Corporate Drive, Bridgewater, NJ 08807, USA; dinesh.simkhada@avantorsciences.com (D.S.); su.teo@avantorsciences.com (S.H.C.T.); nandu.deorkar@avantorsciences.com (N.D.)

**Keywords:** transfection, gene delivery, non-viral vectors, lipid nanoparticles (LNPs), mRNA therapeutics, CAR-T cell therapy, gene therapy, regenerative medicine, siRNA, antisense oligonucleotides (ASOs), gene editing

## Abstract

**Background**: Transfection is vital for gene therapy, mRNA treatments, CAR-T cell therapy, and regenerative medicine. While viral vectors are effective, non-viral systems like lipid nanoparticles (LNPs) offer safer, more flexible alternatives. This work explores emerging non-viral transfection technologies to improve delivery efficiency and therapeutic outcomes. **Methods**: This review synthesizes the current literature and recent advancements in non-viral transfection technologies. It focuses on the mechanisms, advantages, and limitations of various delivery systems, including lipid nanoparticles, biodegradable polymers, electroporation, peptide-based carriers, and microfluidic platforms. Comparative analysis was conducted to evaluate their performance in terms of transfection efficiency, cellular uptake, biocompatibility, and potential for clinical translation. Several academic search engines and online resources were utilized for data collection, including Science Direct, PubMed, Google Scholar Scopus, the National Cancer Institute’s online portal, and other reputable online databases. **Results**: Non-viral systems demonstrated superior performance in delivering mRNA, siRNA, and antisense oligonucleotides, particularly in clinical applications. Biodegradable polymers and peptide-based systems showed promise in enhancing biocompatibility and targeted delivery. Electroporation and microfluidic systems offered precise control over transfection parameters, improving reproducibility and scalability. Collectively, these innovations address key challenges in gene delivery, such as stability, immune response, and cell-type specificity. **Conclusions**: The continuous evolution of transfection technologies is pivotal for advancing gene and cell-based therapies. Non-viral delivery systems, particularly LNPs and emerging platforms like microfluidics and biodegradable polymers, offer safer and more adaptable alternatives to viral vectors. These innovations are critical for optimizing therapeutic efficacy and enabling personalized medicine, immunotherapy, and regenerative treatments. Future research should focus on integrating these technologies to develop next-generation transfection platforms with enhanced precision and clinical applicability.

## 1. Introduction

Transfection is a fundamental technique in biosciences and biotechnology, playing a critical role in developing cell and gene therapies. It enables the introduction of nucleic acids into cells, a crucial step in gene editing, gene therapy, and protein production for drug discovery [1]. Despite its importance, mechanisms governing transfection efficiency remain only partially understood [1,2].

The complex interactions of transfection reagents with cells and how they facilitate genetic material uptake, thereby influencing cellular processes, are actively being studied. While optimized for certain cell types and applications, achieving high transfection efficiency across diverse cell lines, especially primary cells, stem cells, and complex tissues, remains a significant challenge [3]. Furthermore, the toxicity of many transfection reagents is poorly understood, and their potential side effects on cell viability and gene expression limit broader therapeutic utility [2]. Variability in transfection efficiency, the need for specialized reagents, and the complexities of protocol optimization also continue to pose challenges [3,4].

A more informative approach to understanding and placing these limitations into perspective must consider the conventional means of transfection and the practical compromises. The traditional approaches, such as electroporation, gene gun (biolistic), and viral vector delivery, have been the workhorse of gene delivery techniques for decades. Electroporation is the application of high-voltage electrical pulses to form temporary pores in the cell membrane, to which the nucleic acids are delivered. It is effective across various cell types and allows for both transient and stable transfection, but it often results in high cell death and requires cell-type-specific optimization [5,6]. The gene gun approach utilizes high-pressure gas to introduce DNA-covered microparticles into cells and is optimal for plant cells or aggregated tissues but has minimal transfection efficiency and cellular damage potential [7]. Virus vectors, including lentiviruses, adenoviruses, and retroviruses, possess high delivery efficiency and long-lasting gene expression because they become incorporated into the genome of the host. However, these vectors are plagued by significant biosafety and immunogenicity concerns, as well as production and regulatory challenges [8]. While these legacy methods have been instrumental to gene delivery progress, their limitations in terms of safety, consistency, and scalability underscore the imperative for new technologies.

To address these challenges, non-viral gene delivery systems have garnered increasing attention due to their improved safety profiles and engineering tractability [9]. Among these, metal–organic frameworks (MOFs) and inorganic nanoparticles have emerged as outstanding candidates for next-generation platforms. MOFs, composed of metal ions coordinated with organic ligands, exhibit outstanding structural features, such as high porosity, tunable surface chemistry, and biocompatibility [10]. Such features make MOFs highly capable of encapsulating and protecting nucleic acids such as plasmid DNA, siRNA, mRNA, and CRISPR/Cas reagents, while facilitating controlled release and cellular uptake promotion via functionalization. At the same time, inorganic nanoparticles, such as gold nanoparticles, silica nanoparticles, and calcium phosphate-based systems, are highly efficient for nucleic acid delivery [11,12]. They are capable of being engineered to perform surface functionalization for increased endosomal release, improved target specificity, and reduced immunogenicity [9]. MOFs and inorganic nanoparticles are less cytotoxic than cationic polymers and offer convenient platforms for gene targeting in cancer treatment and regenerative medicine [13]. Nevertheless, significant hurdles such as scale-up manufacture, biodegradability, potential toxicity of metal ions, and controlled release kinetics continue to impede clinical translation. Despite these issues, their tunability for physicochemical properties puts MOFs and inorganic nanoparticles in the vanguard of prospective non-viral gene delivery candidates [14].

The limitations of conventional transfection methods—low efficacy in primary and stem cells, cytotoxicity, and variability—highlight the need for innovative and versatile gene delivery systems. The objective of the current review is to comprehensively evaluate existing non-viral transfection methods and systematically compare their efficacy in terms of delivering genetic material and expression and biosafety, including potential for toxicity, immunogenicity, and off-target effects that can be reliably used in research and clinical settings [15,16,17,18]. Furthermore, the review explores and highlight platforms that are pushing the boundaries of gene delivery and assessing enhance therapeutic applications.

## 2. Non-Viral Gene Delivery Systems

Non-viral gene delivery systems offer key advantages over viral vectors, including safety, higher payload capacity, and reduced immunogenicity. Unlike viral vectors, these systems do not integrate genetic material into the host genome [19], minimizing the risk of insertional mutagenesis, enabling transient therapeutic effects—ideal for applications like vaccines. Their higher payload capacity allows delivery of larger or multiple genes, including CRISPR components [20]. Non-viral approaches elicit minimal immune responses, enabling repeated administration and increased safety in immunocompromised patients.

**Figure 1:**
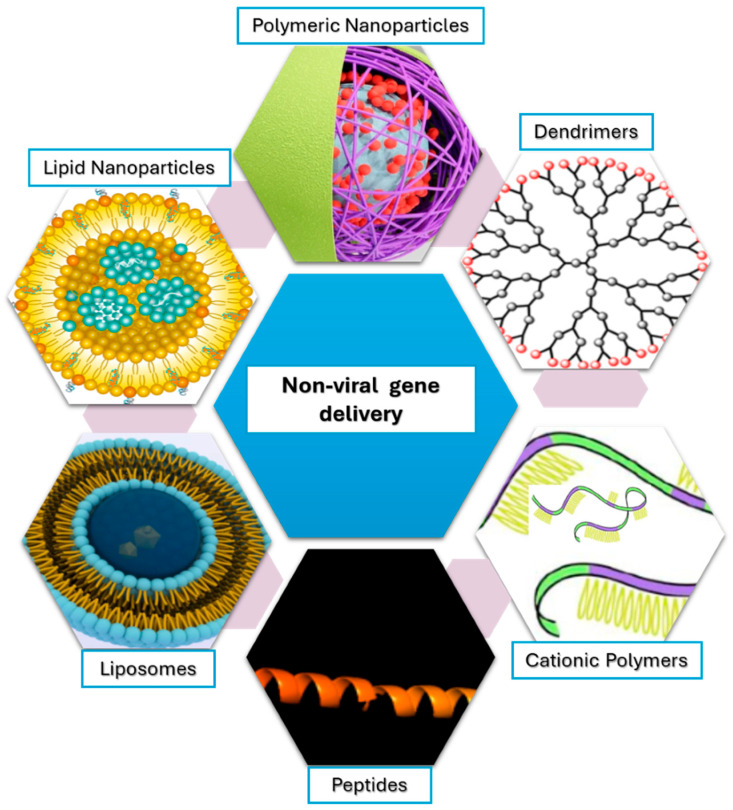
Overview of Non-Viral Gene Delivery: Liposomes: Encapsulate genetic material for delivery; Lipid Nanoparticles: Protect and deliver genes efficiently; Polymeric Nanoparticles: Condense DNA/RNA for delivery; Dendrimers: Branched polymers for gene delivery; Cationic Polymers: Bind and protect genetic material; Peptides: Facilitate targeted gene delivery.

Furthermore, they can deliver diverse genetic material, including DNA, RNA, and oligonucleotides, making them adaptable for gene editing [21,22,23,24,25,26,27], and RNA interference therapies [28]. Common platforms include lipid-based carriers, such as lipoplexes and lipid nanoparticles (LNPs), and cationic polymers, such as polyethyleneimine (PEI) and chitosan, which form polyplexes with nucleic acids. These carriers protect genetic cargo, aid in cellular uptake and endosomal escape, and can be engineered for tissue-specific delivery. In short, non-viral systems are economical, scalable, and compatible with targeted delivery to specific tissues (Figure 1).

Extracellular vesicles (EVs), especially exosomes, are emerging as powerful non-viral vectors for gene delivery due to their natural origin, biocompatibility, and ability to facilitate intercellular communication. These nano-sized vesicles (30–150 nm) protect genetic cargo, such as DNA, siRNA, and mRNA, from enzymatic degradation, thereby improving delivery stability and efficiency. Their low immunogenicity and toxicity make them suitable for repeated use, even in immunocompromised patients [29,30]. Exosomes can be engineered either post-isolation (e.g., electroporation) or via donor cell modification to load nucleic acids endogenously. Surface modifications further enable targeted delivery [31]. Despite challenges in large-scale production and standardization, their biomimetic properties make exosomes promising candidates for RNA therapeutics and precision medicine [32,33].

These combined advantages position non-viral delivery as a key innovation in driving the development of therapeutic gene editing technologies [34,35]. This potential is reflected in the active engagement of numerous pharmaceutical companies in developing therapeutic drugs targeting diverse diseases using non-viral gene delivery systems (Table 1). By leveraging these systems, these companies aim to enhance the efficiency and targeting of therapeutic gene delivery, ultimately improving treatment outcomes and broadening the clinical applications of gene therapy [36].

## 3. Advancing to Clinical Path Through Transfection

**Figure 2:**
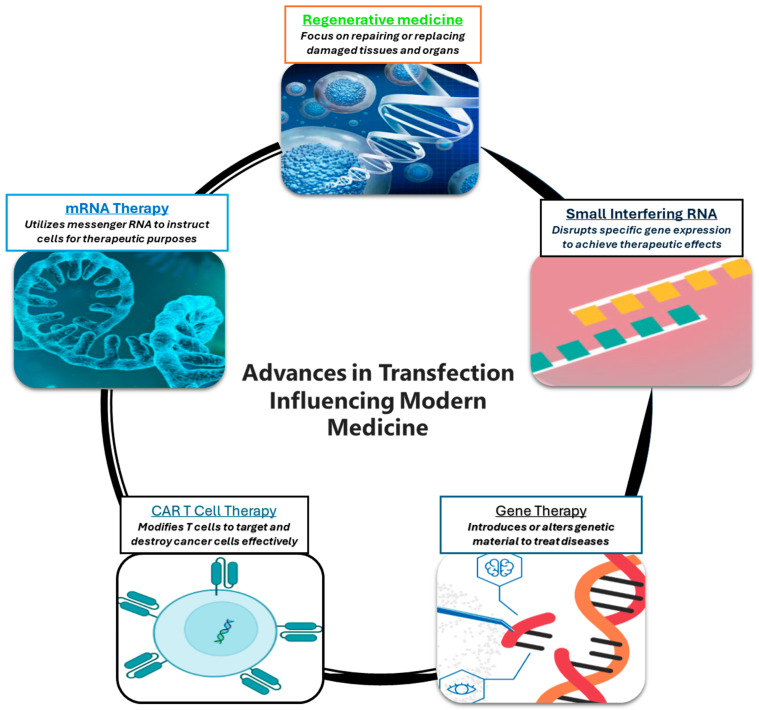
Transfection Driving Therapeutic Development: mRNA-Based Therapies: Instruct cells to produce therapeutic proteins; CAR-T Cell Therapy: Modify T cells to target cancer; Gene Therapy: Alter genes to treat disorders; siRNA: Silence genes to prevent harmful proteins; Regenerative Medicine: Promote tissue repair and regeneration.

Transfection technologies are significantly improving clinical applications through innovative approaches leading to the development of new therapies and optimizing existing treatments (Figure 2).

### 3.1. mRNA-Based Therapies

The field of mRNA therapeutics has rapidly advanced due to its potential in addressing various medical conditions. Unlike traditional protein therapies, mRNA enables in vivo protein production, bypassing complex purification processes [56,57]. The rapid development and deployment of COVID-19 vaccines exemplifies the power of mRNA-based vaccines [58,59,60,61]. Beyond vaccines, mRNA is being explored for protein-replacement therapy [57], cancer immunotherapy, cellular reprogramming, and gene editing [62,63,64].

Effective mRNA-based therapies depend on efficient transfection technologies. Delivering mRNA to target cells is challenging due to its susceptibility to degradation and the need for suitable carriers to facilitate intracellular uptake [60,65]. Several transfection methods have been explored, including lipid-based systems, polymer-based systems, and viral transduction, each with advantages and limitations. Polymers, especially cationic polymers, offer reduced immunotoxicity, low cost, and ease of production, but face challenges with low transfection efficiency and potential toxicity [66]. While viral transduction is highly efficient for delivering genetic material into hard-to-transfect cells, including primary cells, it carries the risk of increased cytotoxicity and viral infection [67,68].

Lipid nanoparticles (LNPs) have emerged as the leading non-viral delivery platform, showing great promise for in vivo delivery of RNA therapeutics, such as small interfering RNAs (siRNA) [69], antisense oligonucleotides (ASOs), synthetic guide RNAs [70,71], and mRNAs [72,73]. LNPs offer flexibility in payload adaptation and lipid composition, making them a transformative tool in drug delivery [74,75]. Their stability, efficiency, and reduced immunogenicity position them pivotal in advancing vaccines, gene therapies, and antibody-based treatments.

### 3.2. CAR-T Cell Therapy

CAR-T cell therapy represents a significant advance in cancer treatment. It involves genetically engineered T cells which possess a remarkable ability to target and destroy cancerous cells with high specificity [76,77]. CAR-T cells have demonstrated impressive results against hematological malignancies such as, acute lymphoblastic leukemia (ALL) and large B-cell lymphoma, offering potentially curative outcomes for patients unresponsive to conventional treatments [78]. This therapy relies on advanced transfection technologies to precisely modify T cells enabling them to express chimeric antigen receptors (CARs) [79,80]. While viral vectors, like lentiviruses and retroviruses, are commonly used for transduction due to their efficient and stable and transfer of CAR genes into T cells [81], they carry a potential of insertional mutagenesis due to integration of genetic material into the host genome.

Beyond viral vectors, non-viral approaches, such as electroporation, have emerged as attractive alternatives that allow rapid and inexpensive introduction of CAR-encoding mRNA or DNA into T cells, thus largely avoiding integration-related risks [82,83]. Furthermore, polymer-based and lipid nanoparticle (LNP) systems are also being explored as safer, less immunogenic methods that also facilitate scalable CAR-T cell production [84]. These advances in transfection technology are not only improving the efficiency of CAR-T therapy but also expanding its application to solid tumors, which pose unique challenges such as antigen heterogeneity and limited T cell infiltration [85,86,87,88]. These engineered cells may possess dual-targeting capabilities and resistance to tumor-induced immunosuppression. Innovations like electroporation and RNA-based CAR transfection are streamlining manufacturing processes, lowering costs, and increasing flexibility. Collectively, these advances enhance the safety and efficacy of CAR-T therapy and broaden its clinical applicability to a wider range of cancers [87,88].

### 3.3. Revolutionizing Gene Therapy

Gene therapy has emerged as a revolutionary modality in modern medicine, offering the potential to treat or even cure genetic disorders by directly targeting the underlying genetic defects. A critical component of successful gene therapy is the development and optimization of transfection technologies which enable the safe and efficient delivery of therapeutic genes into target cells [89,90,91]. Beyond monogenic disorders, gene therapy is also contributing to advances in cancer treatment through the genetic engineering of immune cells to enhance tumor suppression and immune response [92,93]. These advances are often enabled by transfection methods such as electroporation and nanoparticle delivery. Furthermore, innovations in in vivo delivery approaches—including adaptations of lipid nanoparticle (LNP) technology from mRNA vaccines and tissue-specific targeting via receptor-mediated pathways—are addressing significant challenges, improving precision, and expanding the therapeutic potential of gene therapy [94,95]. Continued evolution of transfection technologies is focused on overcoming challenges related to efficiency, immunogenicity, and scalability [96]. These ongoing developments are poised to transform the treatment landscape for a range of diseases, including genetic disorders, cancer, and other complex conditions [97,98,99].

### 3.4. Small Interfering RNA and Antisense Oligonucleotide Therapies

Small interfering RNA (siRNA) and antisense oligonucleotides (ASOs) represent powerful gene silencing and therapeutic gene-modifying tools with significant potential for treating genetic disorders, viral infections, and cancers. Effective delivery to target tissues is crucial for their therapeutic success, and this relies heavily on optimized transfection reagents [100,101,102,103]. siRNA therapies, exemplified by the FDA-approved Onpattro for hereditary transthyretin-mediated amyloidosis, use RNA interference to degrade mRNA and require LNPs for precise targeting [100,104]. In contrast, ASOs, such as Spinraza for spinal muscular atrophy (SMA) and Eteplirsen for Duchenne muscular dystrophy (DMD), bind to mRNA to inhibit translation or correct splicing defects [105]. Advances in cationic lipids and polymers have led to improved ASO delivery, enhancing stability, targeting specificity and minimizing immune responses [106,107,108,109,110,111,112,113,114]. These innovations in delivery technologies are expanding the scope of RNA-based therapeutics, offering new hope for patients with previously untreatable genetic disorders [114,115,116,117,118,119,120,121].

### 3.5. Regenerative Medicine

The efficacy of regenerative medicine strategies, including stem cell therapy, tissue engineering, and gene therapy, is significantly enhanced by advances in transfection technologies [122]. A pivotal advance in therapies is the utilization of transfection reagents, which markedly enhance gene delivery efficiency. These techniques facilitate precise genetic modifications, driving tissue repair and cellular regeneration. In stem cell therapy, electroporation, viral vectors, and nanoparticle-based delivery systems are pivotal for introducing gene-editing tools, such as CRISPR/Cas9, enabling targeted genomic modifications and optimizing therapeutic efficacy [123,124,125,126].

Tissue engineering utilizes transfection-mediated delivery of growth factor genes to promote the regeneration of complex tissues, including bone, cartilage, and nerves. Notably, mRNA-lipid nanoparticle systems offer a non-viral, low-risk approach for cellular reprogramming, enabling the generation of specialized cell types [127]. Gene therapy employs these transfection technologies to deliver therapeutic genes, addressing conditions like ischemic injuries, muscular dystrophy, and retinal degeneration). These methodologies feature the critical role of efficient gene delivery in realizing the full potential of regenerative medicine [128]. Examples of specific therapies developed by pharmaceutical companies that utilize non-viral gene delivery systems are summarized in Table 2.

## 4. Key Challenges in Transfection

**Figure 3:**
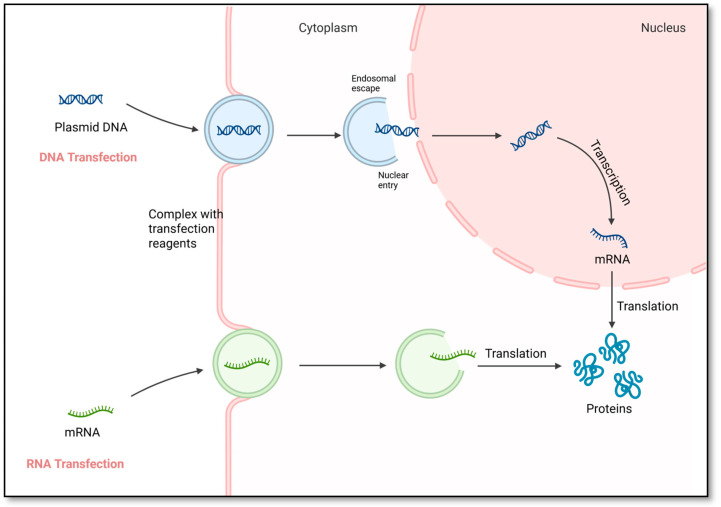
Non-Viral DNA Transfection: This illustration depicts the gene transfection processes, effectively navigating various obstructions to deliver nucleic acids. [Created in BioRender. Teo, C. (2025) https://BioRender.com/ludvsnm, premium version] [164].

The field of gene delivery has witnessed substantial progress with the development of sophisticated transfection technologies. These include liposomal, polymer-based systems, offering biocompatibility and controlled release; viral vectors, providing high transduction efficiency; nanoparticle-mediated delivery, enabling targeted gene transfer; electroporation, facilitating direct cellular uptake; and microfluidic approaches, allowing for precise control and high-throughput applications [136,137]. Each of these methods present unique advantages and pose specific challenges [138,139] associated with efficient and safe gene delivery (Figure 3).

Chemical transfection, a widely employed method, utilizes cationic reagents, including lipids and polymers to complex with nucleic acids. This facilitates cellular entry primarily through endocytosis [140,141]. Lipofection, a prominent technique within this category, leverages cationic lipids to form lipoplexes, enabling efficient transmembrane transport. While alternative reagents such as calcium phosphate and DEAE-dextran have been utilized, they generally exhibit lower transfection efficiency and increased cytotoxicity [2,142].

Cationic polymers—such as polyethyleneimine (PEI), are employed in polymer-based transfection to form nanoparticles that deliver nucleic acids into cells [143,144,145]. However, the use of these polymers has been historically limited by concerns of their inherent cytotoxicity and biocompatibility. To address these limitations, the development of biodegradable and biocompatible polymers, such as poly (lactic-co-glycolic acid) (PLGA) and poly(beta-amino esters) (PBAEs), has significantly improved the safety profile and transfection efficiency, especially in in vivo settings [146].

Exosome-based gene delivery shows great potential, but several challenges hinder its clinical translation. Standardizing and scaling production remain difficult due to inconsistent yields and purity, which complicates reproducibility and quality control [32]. Cargo loading methods often compromise efficiency or vesicle integrity, and while exosomes offer natural targeting, achieving precise tissue-specific delivery without complex surface engineering is still technically demanding [30,147]. Additionally, limited understanding of cellular uptake and intracellular trafficking reduces the predictability of gene expression outcomes [29]. The absence of standardized protocols and regulatory frameworks further delays clinical adoption [148]. Addressing these issues is key to realizing exosomes as scalable, safe, and effective non-viral delivery systems.

Physical transfection methods, including electroporation and microinjection, offer direct nucleic acid delivery by circumventing cellular barriers [149]. Electroporation utilizes electrical impulses to induce transient membrane permeabilization, facilitating nucleic acid uptake, while microinjection enables precise, single-cell delivery [150]. Despite their efficacy in specialized applications, these techniques are limited by scalability, potential cellular damage, and reduced cell viability [151]. A significant challenge across all transfection modalities is endosomal trapping, wherein nucleic acids are sequestered within endosomes, hindering their functional activity [152,153,154]. To address this, substantial efforts have focused on enhancing endosomal escape through the development of fusogenic lipids, pH-responsive materials, and other escape enhancers, leading to improved delivery efficiency [136,155,156,157].

Despite promising preclinical results, metal–organic frameworks (MOFs) and inorganic nanoparticles face significant hurdles in reaching clinical use. While these materials offer advantageous properties like tunable porosity, high surface area, and chemical functionalization, most MOFs are still in early research and development [158,159]. Several critical issues impede their transition into clinical applications, such as drug delivery and bioimaging. These include limited biocompatibility data, potential long-term toxicity, challenges in large-scale synthesis, and regulatory ambiguities [159]. Overcoming these challenges is crucial to fully harness the therapeutic potential of MOFs and other inorganic nanomaterials [160].

Microfluidic technologies leverage precise control of fluid flow at the microscale to introduce genetic material into cells with high accuracy [161]. These systems offer significant benefits, including minimized reagent consumption, precise spatiotemporal control of transfection parameters, and the capability for single-cell manipulation This makes them advantageous for high-throughput applications and for specialized techniques such as intracellular delivery and mechanoporation [162]. However, the mechanical and physical forces generated within microfluidic devices, particularly shear stress, can induce cellular stress, potentially leading to compromised cell viability and reduced transfection efficiency [163].

Nucleic acid delivery faces significant obstacles, including extracellular degradation, serum interactions, and intracellular membrane penetration [165]. Physical methods, while aiding cellular entry, can compromise cell viability, and viral vectors pose inherent safety risks. Non-viral synthetic vectors, notably lipid-based systems, offer a safer approach but necessitate optimization to enhance gene delivery efficiency [166]. Their clinical translation is hindered by tropism-related challenges. These systems lack the intrinsic targeting specificity of viral vectors, which naturally engage receptors on target cells [167]. To compensate, non-viral carriers often require surface functionalization with ligands, antibodies, or peptides to achieve tissue-specific delivery [167,168]. However, receptor heterogeneity among cell types complicates consistent targeting, leading to off-target distribution and variable therapeutic efficacy [167]. Additionally, functionalization efforts can impair vector stability, reduce uptake efficiency, or trigger rapid clearance by the mononuclear phagocyte system (MPS) or reticuloendothelial system (RES) [167,169]. The prevalent liver tropism of many nanoparticle systems further limits delivery to other organs unless physical or molecular targeting strategies are employed [170,171]. Thus, achieving precise tropism without sacrificing biocompatibility, efficiency, or scalability remains a critical and unsolved challenge for the effective use of non-viral gene delivery systems in clinical settings [172].

As another example, hematopoietic stem cell (HSC) transfection systems can be considered for tissue-specific tropism. Transfecting HSCs remains a formidable challenge due to their quiescent nature, sensitivity to manipulation, and the need to preserve their self-renewal and multilineage differentiation potential [173]. However, the emergence of non-viral transfection technologies has significantly enhanced the feasibility of genetic modification in these cells, offering safer, more scalable, and less immunogenic alternatives to traditional viral vectors. Among these, ribonucleoprotein (RNP) complex delivery has emerged as a particularly promising strategy. This method involves the direct introduction of pre-assembled CRISPR-Cas9 protein complexes with guide RNA into HSCs, enabling rapid and highly specific genome editing while circumventing the risks of insertional mutagenesis associated with DNA-based approaches [172]. Recent technological advancements have further refined RNP delivery through innovative platforms such as microfluidics, which allow precise modulation of transfection parameters; filtroporation, which employs pressure gradients to facilitate cytosolic entry; and cell-penetrating peptides (CPPs), which enhance membrane permeability with minimal cytotoxicity. Collectively, these developments are transforming the landscape of HSC engineering, paving the way for safer and more effective gene therapies for hematologic and genetic disorders [174].

## 5. Transfection Technologies: Future Perspectives

The next generation of transfection technologies aims to improve efficiency, reduce immunogenicity, and enable precise gene delivery [59,175,176]. Specifically, mRNA-based therapies face obstacles such as instability, immune activation, and limited tissue targeting. To overcome these challenges, strategies involving ionizable lipids, fusogenic materials, dendrimers, and biodegradable polymers have been implemented to increase safety and broaden therapeutic applicability for clinical translation [177,178,179,180,181,182,183,184,185]. LNPs can be engineered for targeted gene delivery by manipulating their chemical composition and charge, as evidenced by successful CRISPR/Cas9-mediated PTEN editing. The inclusion of ionizable cationic lipids in LNP formulations is essential for encapsulating CRISPR/Cas9 components, enhancing the circulation half-life, and improving endocytosis efficiency by target cells [186]. Although LNPs are beneficial in terms of scalability and the ability to deliver large molecules, extracellular vesicles (EVs) are being explored as biocompatible and nonantigenic delivery vehicles for mRNAs [187]. EVs can currently be loaded with mRNA via specialized plasmids or direct methods [188]. Moreover, EVs loaded with targeting peptides, such as those that use the C1C2 domain of lactadherin, have been found to be effective in preclinical cancer therapy with prolonged circulation and no toxicity [189]. LNPs and EVs are suitable candidates for gene therapy, with LNPs being more potent in large-scale applications and EVs having improved biocompatibility and lower toxicity.

CPPs also show promise as effective vectors for mRNA delivery with high cellular uptake and endosomal escape efficacy. PepFect14 (PF14), for example, is used to create stable nanocomplexes for promoting mRNA delivery [190], whereas RALA is a pH-sensitive peptide used in cancer treatment [191]. The HIV-1 Tat peptide has been extensively used in mRNA vaccine design and gene expression research [192]. Moreover, MPG has shown potential in regenerative medicine [193], whereas CADY has high transfection efficiency for therapeutic purposes [194]. Overall, the development of next-generation delivery systems aims to address several critical challenges, including low transfection efficiency in difficult-to-transfect cell types, high cytotoxicity, poor compatibility with high-throughput screening (HTS) platforms, inconsistent reproducibility, and limited formulation stability during storage. Overcoming these barriers is crucial for advancing molecular and cellular biology and for enabling translational applications such as gene therapy and regenerative medicine.

In CAR-T cell therapy, CAR-T cells modified with the Sleeping Beauty (SB) transposon system have demonstrated potent antileukemic activity with a favorable safety profile. This non-viral approach reduces the risk of insertional mutagenesis, lowers manufacturing cost and simplifies production. SB-engineered CAR-T cells effectively target leukemia and are being investigated for allogeneic CAR-T cell therapies [40]. Since 2018, LNP-based manufacturing strategies have been rigorously investigated to optimize CAR-T cell production. Early studies demonstrated a reduction to a 3-day manufacturing timeline, with recent advances utilizing activating lipid nanoparticles achieving a remarkable 1-day production cycle [38]. This process acceleration, facilitated by innovative LNP formulations, enables scalable production of autologous therapies by streamlining workflows rather than simply increasing volume. The integration of closed systems further enhances manufacturing efficiency by eliminating bead-mediated T cell activation, simplifying culture procedures, and reducing costs, thereby bolstering centralized production capacity [39]. These improvements are particularly significant for patients with rapidly progressing cancers, where swift treatment access is essential [37].

The future of transfection technologies is poised between biotechnology and artificial intelligence (AI) in the pipeline, with the promise of transforming gene therapy and regenerative medicine. AI-driven modeling is demystifying the complexity of intricate cellular interactions, enabling rational design of the next generation of gene delivery systems with more precision, less toxicity, and cell-type specificity [195,196]. By leveraging machine learning and computational simulations, researchers can now predict optimal transfection conditions, streamline vector engineering, and minimize the traditional reliance on trial-and-error experimentation. AI algorithms have already shown to be successful for the optimization of lipid nanoparticle (LNP) formulations for mRNA delivery, simulation of nucleic acid–polymer interactions, and prediction of physicochemical properties of novel nanocarriers, such as polyplexes and MOF-based platforms [197,198]. In silico technologies also enable the virtual screening of carrier libraries to improve cellular uptake, endosomal escape, and intracellular stability—key determinants of effective transfection [199]. This foresight role not only accelerates the experimental process but also bridges the translational gap between the research bench and clinic. Moreover, the integration of AI with single-cell transcriptomics and omics data allows for personalized gene delivery approaches via the identification of unique molecular signatures and tissue-specific receptors [200]. The confluence of computational modeling and lab validation holds the potential to accelerate regulatory approval pathways, reduce development expenditure, and enhance patient access to novel gene therapies. With the advancement of AI, its role in guiding non-viral vector design, dosage optimization, and therapeutic targeting will play a pivotal role in shaping the future of precision medicine.

Artificial intelligence (AI) is transforming the optimization of transfection efficiency in lipid nanoparticle (LNP)-mediated mRNA delivery by leveraging deep learning, multimodal data fusion, and explainable AI [87,201,202]. Models like TransMA integrate 3D molecular geometry with 1D atomic sequences to deliver high accuracy and interpretable attention maps, aiding in the identification of transfection cliffs [198]. LANTERN combines Morgan fingerprints with expert descriptors in a multilayer perceptron, outperforming complex models like AGILE with strong generalizability [198]. TransLNP, a transformer-based model using the BalMol data-balancing technique, excels in predicting across diverse LNPs and small datasets [203]. Meanwhile, AGILE uses a graph neural network trained on 60,000 virtual lipids for effective large-scale screening (AGILE platform) [204]. Collectively, these AI tools are reshaping mRNA delivery by enabling more efficient and scalable formulation strategies [205,206].

## 6. Conclusions

Transfection technologies continue to evolve, addressing efficiency, safety, and scalability concerns to unlock their full potential in gene therapy, drug development, and disease treatment. As transfection methodologies advance, they promise to revolutionize the landscape of biomedical sciences by enhancing gene therapy, drug discovery, and cellular engineering. Emerging techniques, such as lipid nanoparticle systems and non-viral gene delivery, provide innovative solutions to existing challenges, optimizing safety and efficacy across applications. The interplay between efficiency, toxicity, and scalability is driving continuous innovation in gene delivery, with advances in lipid nanoparticles, non-viral vectors, and electroporation providing promising alternatives. By addressing current limitations, researchers are not only improving transfection methodologies but also expanding the therapeutic possibilities of genetic medicine, bringing new hope for precision treatments. In closing, this review provides a strategic roadmap for researchers working on gene delivery technologies, especially those bridging research with therapeutic development and clinical application.

## Figures and Tables

**Table 1:** Non-viral gene delivery systems in clinical trials [37,38,39,40,41,42,43,44,45].

Description	Applications	Clinical Trials (ClinicalTrials.gov)	References
**Liposomes**	Targeted cancer therapy-Solid Tumors	NanoLiposome-Phase 1 (NCT02834611 | Card Results | ClinicalTrials.gov)	Keystone Nano [46,47]
Lipid Nanoparticles (LNPs)	mRNA-based enzyme replacement	ARCT-810-Phase 1 (NCT04416126 | Card Results | ClinicalTrials.gov) ARCT-032-Phase 2 (NCT06747858 | Card Results | ClinicalTrials.gov)	Arcturus Therapeutics [48,49]
	Infectious disease vaccine-COVID-19 mRNA Vaccine	ARCT-021-Phase 2 (NCT04480957 | Card Results | ClinicalTrials.gov)	
Polymeric Nanoparticles	Radiation enhancer for cancer	NBTXR3-Phase 1/2/3 (NCT05039632 | Card Results | ClinicalTrials.gov)	Nanobiotix [50]
	Tumor-targeted chemotherapy	CRLX101-Phase 1/2a (NCT02187302 | Card Results | ClinicalTrials.gov)	Lumos Pharma [51]
	Nanoparticle drug delivery-Triple-negative Breast Cancer, Small Cell Lung Cancer	NK012-Phase 2 (NCT00951054 | Card Results | ClinicalTrials.gov)	Nippon Kayaku [52]
Dendrimers	Cancer therapy	DEP SN38: (Clinical Trials register—Search for DEP SN38)	StarPharma [53]
Cationic Polymers (e.g., PEI)	Antimicrobial therapy	Brilacidin-Phase 2a (NCT02052388 | Card Results | ClinicalTrials.gov)	Innovation Pharmaceuticals [54]
Peptide	Metastatic tumors	177Lu-Integrin-Phase 1 (Study Details | Study to Evaluate the Safety and Activity (Including Distribution) of 177Lu-3BP-227 in Subjects with Solid Tumors Expressing Neurotensin Receptor Type 1. | ClinicalTrials.gov)	PeptiDream [55]

**Table 2:** Therapies using Non-Viral Gene Delivery Systems [129,130,131,132,133,134,135].

Category	Company	Drug	Description
mRNA-based Therapies	Moderna	mRNA-1273	COVID-19 vaccine using lipid nanoparticles for delivery.
	Pfizer-BioNTech	BNT162b2	COVID-19 vaccine using lipid nanoparticles for delivery.
	Arcturus Therapeutics	ARCT-810	mRNA therapy for Ornithine Transcarbamylase (OTC) deficiency using lipid nanoparticles.
	CureVac	CVnCoV	COVID-19 vaccine candidate using lipid nanoparticles.
	Translate Bio (Sanofi)	MRT5005	mRNA therapy for cystic fibrosis using lipid nanoparticles.
	BioNTech	BNT111	mRNA cancer immunotherapy using lipid nanoparticles.
	GenEdit	Various	Developing non-lipid, hydrophilic polymer nanoparticles for autoimmune diseases and other indications.
CAR-T Therapies	Cellectis	UCART19	Allogeneic CAR-T therapy for leukemia using TALEN gene editing technology.
	Poseida Therapeutics	P-BCMA-101	CAR-T therapy for multiple myeloma using piggyBac DNA Modification System.
	Precision BioSciences	PBCAR0191	CAR-T therapy for B-cell malignancies using ARCUS genome editing technology.
	Sana Biotechnology	SC291	CAR-T therapy for hematologic malignancies using fusogen technology for non-viral delivery.
	Allogene Therapeutics	ALLO-501	Allogeneic CAR-T therapy for non-Hodgkin lymphoma using TALEN gene editing technology.
	Arcellx	CART-ddBCMA	CAR-T therapy for multiple myeloma using a novel synthetic binding scaffold.
Gene therapy	ElevateBio	Various	Developing a broad portfolio of cell and gene therapies using non-viral delivery systems.
	Tessera Therapeutics	Gene Writing™	Pioneering Gene Writing technology to treat diseases at their source.
	Mediphage Bioceuticals	ministring DNA (msDNA)	Developing non-viral, safe, and redosable gene therapies using msDNA technology.
	Clearside Biomedical	CLS-AX	Developing therapies for chronic eye diseases using non-viral delivery methods.
	Code Biotherapeutics	3DNA	Leveraging a non-viral multivalent synthetic DNA delivery platform for various genetic disorders.
	Mana.bio	Various	Using AI-based drug delivery platform for oligonucleotide therapies, including mRNA-based therapeutics.
	Nanoscope Therapeutics	MCO-010	Developing gene therapies for vision impairment and blindness using non-viral delivery systems.
	Generation Bio	ceDNA	Developing non-viral genetic medicines with long-term efficacy and support for redosing.
siRNA and ASO Therapies	Alnylam Pharmaceuticals	Onpattro (patisiran)	siRNA therapy for hereditary transthyretin-mediated amyloidosis using lipid nanoparticles.
	Ionis Pharmaceuticals	Spinraza (nusinersen)	ASO therapy for spinal muscular atrophy.
	Arrowhead Pharmaceuticals	ARO-AAT	siRNA therapy for alpha-1 antitrypsin deficiency using TRiM™ platform.
	Dicerna Pharmaceuticals	DCR-PHXC	siRNA therapy for primary hyperoxaluria using GalXC™ platform.
	Wave Life Sciences	WVE-120101	ASO therapy for Huntington’s disease using stereopure oligonucleotides.
	Silence Therapeutics	SLN360	siRNA therapy for cardiovascular disease using GalNAc conjugation.
	ProQR Therapeutics	Sepofarsen	ASO therapy for Leber congenital amaurosis 10 (LCA10).
	Arbutus Biopharma	AB-729	siRNA therapy for chronic hepatitis B using GalNAc conjugation.
	OliX Pharmaceuticals	OLX101	siRNA therapy for hypertrophic scars using asymmetric siRNA technology.
	DTx Pharma	FALCON platform drugs	Developing siRNA therapies using Fatty Acid Ligand Conjugated OligoNucleotide (FALCON) platform.
Regenerative Medicine	Aspen Neuroscience	ANPD001	Autologous iPSC-derived neuron replacement therapy for Parkinson’s Disease.
	Nanoscope Therapeutics	MCO-010	Gene therapy for vision impairment and blindness using non-viral delivery systems.
	Generation Bio	ceDNA	Developing non-viral genetic medicines with long-term efficacy and support for redosing.
	Tessera Therapeutics	Gene Writing™	Pioneering Gene Writing technology to treat diseases at their source.
	Code Biotherapeutics	3DNA	Leveraging a non-viral multivalent synthetic DNA delivery platform for various genetic disorders.
	Clearside Biomedical	CLS-AX	Developing therapies for chronic eye diseases using non-viral delivery methods.

## Data Availability

All data and materials utilized in this study are sourced exclusively from publicly available databases and published research. As outlined in the references, the information has been obtained from open-access repositories and previously published works, ensuring transparency and accessibility. No proprietary or restricted datasets have been used, and all referenced materials are freely accessible to the public, allowing for independent verification and further analysis by other researchers.

## Abbreviations

AI	Artificial intelligence
ALL	Acute lymphoblastic leukemia
ASOs	Antisense Oligonucleotides
CAR-T	Chimeric antigen receptor-T
COVID-19	Coronavirus disease 2019
CPP	Cell penetrating peptides
CRISPR	Clustered regularly interspaced short palindromic repeats
DMD	Duchenne muscular dystrophy
DNA	Deoxy ribonucleic acid
EVs	Extracellular vesicles
HTS	High-throughput screening
HSC	Hematopoietic stem cell
LNPs	Lipid Nanoparticles
MOF	Metal–organic frameworks
mRNA	Messenger RNA
PBAEs	Poly(beta-amino esters)
PEI	Polyethylenimine
PLGA	Poly (lactic-co-glycolic acid)
RNA	Ribonucleic acid
SB	Sleeping Beauty
SiRNA	Small interfering RNA
SMA	Spinal muscular atrophy
